# Selection criteria for radiofrequency ablation for colorectal liver metastases in the era of effective systemic therapy: a clinical score based proposal

**DOI:** 10.1186/1471-2407-14-500

**Published:** 2014-07-09

**Authors:** Axel Stang, Karl Jürgen Oldhafer, Hauke Weilert, Handan Keles, Marcello Donati

**Affiliations:** 1 Department of Hematology and Oncology, Asklepios Hospital Barmbek, Rübenkamp 220, 22291 Hamburg, Germany; 2 Department of Surgery, Asklepios Hospital Barmbek, Rübenkamp 220, 22291 Hamburg, Germany; 3 Department of Hematology and Oncology, Asklepios Hospital Altona, Paul-Ehrlich Straße 1, 22763 Hamburg, Germany; 4 Department of Surgery General and Oncologic Unit, Vittorio-Emanuele University Hospital, 95123 Catania, Italy

**Keywords:** Colorectal cancer, Liver metastases, Radiofrequency ablation, Multimodality treatment, Prognostic factors, Clinical score

## Abstract

**Background:**

At present, there are no widely accepted criteria for the use of radiofrequency ablation (RFA) for the treatment of colorectal liver metastases (CLM) in the context of effective modern-agent therapies. We aimed to define selection criteria for patients with liver-limited CLM who may benefit from adding RFA to systemic therapy with respect to long-term disease control.

**Methods:**

Between 2002 and 2007, 88 consecutive patients received RFA for liver-only CLM during partial remission (PR), stable disease (SD), or progressive disease (PD) after systemic therapy. At a median follow-up of 8.2 years (range 5.2-11.1 years), clinical data were correlated to overall survival (OS) and recurrence-free survival (RFS).

**Results:**

Poor OS and RFS correlated significantly with PD to systemic therapy before RFA (HR 5.46; p < 0.0001; and HR 6.46; p < 0.0001), number of ≥4 CLM (HR 3.13; p = 0.0005; and HR 1.77; p = 0.0389), and carcinoembryonic antigen (CEA) level of ≥100 ng/ml (HR 1.67; p = 0.032; and HR 1.67; p = 0.044). The presence of four criteria (PR, ≤3 CLM, ≤3 cm maximum size, and CEA ≤100 ng/ml) selected a subgroup (n = 23) with significantly higher probabilities for OS and RFS at 5 years (39% and 22%,respectively) compared to those without any or up 3 of these criteria (0-27% and 0-9%, p < 0.001, respectively).

**Conclusions:**

A score based on four criteria (response to systemic therapy, ≤3 CLM, ≤3 cm size, low CEA value) may allow to select patients with liver-only CLM for whom additional use of RFA most likely adds benefit in an attempt to achieve long-term disease control. Almost one-fourth of patients fulfilling these four criteria may achieve 5-year survival without disease recurrence following effective systemic plus local RFA treatment.

## Background

Hepatic resection is the only curative treatment for patients with colorectal liver metastases (CLM), with reported 5-year survival rates ranging from 35 to more than 50% [1,2]. However, 80% of patients are not surgical candidates because of advanced disease and/or comorbidities and receive palliative systemic therapy. Despite the fact that modern-agent regimens consisting of 5-fluorouracil, leucovorin plus oxaliplatin (FOLFOX) or irinotecan (FOLFIRI) ± cetuximab and/or bevacizumab achieve response rates up to 70% [3], complete pathological and/or durable clinical response of CLM is rare [4,5], and patients will typically relapse with decreasing efficacy with each subsequent line of treatment [6,7]. Therefore, long-term (5-year) overall survival (OS) and/or recurrence-free survival (RFS) based on systemic therapy of CLM alone is uncommon [8,9].

Radiofrequency ablation (RFA) has become a widely used local therapy for unresectable CLM [10,11]. Several cohort studies have reported 5-year OS rates of 15-48% after RFA for liver-only CLM [12-18]. Although these results are influenced by selection bias, they consistently indicate that 5-year OS is possible in numerous patients. However, because all these studies report few, if any, data on the use and/or efficacy of systemic therapies, little is known about their impact on clinical outcomes in the RFA collective. Moreover, RFA is an additive, than an alternative, to systemic therapy, which is the standard for care. Despite this, to date, no widely accepted criteria exist for the use of RFA for treatment of CLM in the context of effective modern-agent therapies.

The recently published randomized phase II CLOCC trial supports an impact on disease control by adding RFA to chemotherapy for patients with liver-only CLM [19]. However, data on the long-term efficacy of this combined approach are still limited, namely with respect to 5-year RFS results. Moreover, the importance of response to systemic treatment is still unclear, albeit response may impact on the pattern of use and timing of RFA treatment. Also, patient inclusion criteria in the CLOCC trial (up to 9 CLM of ≤4 cm in size) cover a prognostically heterogeneous group of patients and may not match the optimal candidates for attaining complete local and long-term disease control by RFA treatment. These patients have to be better identified, and their characteristics would be of value for both clinical and research settings.

In the present study, we correlated clinical variables to OS and RFS in 88 consecutive patients with >5 years of follow-up after receiving RFA for liver-only CLM following systemic therapy. Special emphasis was directed to the importance of response to systemic therapy and the characteristics of 5-year survivors without disease recurrence. We attempted to develop a prognostic factor-based score for predicting probabilities of OS and RFS in the pre-RFA setting. The aim was to define selection criteria for patients with liver-limited CLM who may benefit from adding RFA to systemic therapy with respect to long-term disease control.

## Methods

### Study design

We carried out a retrospective analysis of a prospectively recorded database in a single institution with systematic review of patients with potential of at least 5 years of follow-up. The study was approved by the Institutional Review Board "Clinical Ethics Committee" of the Asklepios Hospital Altona. All patients provided written informed consent for data collection and for scientific evaluation of the data.

### Patient cohort

Between January 2002 and December 2007, a total of 88 consecutive patients underwent RFA of CLM after treatment with systemic therapy (combination chemotherapy ± bevacizumab or cetuximab) at the Asklepios Hospital Altona. Inclusion criteria for RFA treatment were: histologically confirmed colorectal adenocarcinoma, ≤5 unresectable liver-only CLM of ≤5 cm maximum size, and anticipated life expectancy of ≥6 months. Unresectability was decided in a multidisciplinary staff meeting, including hepatobiliary surgeons, radiologists, and oncologists. Reasons for unresectability were: technical impossibility to achieve R0 resection with preservation of ≥30% liver parenchyma (e.g. proximity of CLM to the portal vein and/or CLM involving or abutting the vena cava, a major hepatic vein branch, or 2 hepatic veins), contraindications to general anaesthesia (deterioration of general condition and/or cardiorespiratory disease), and patient refusal. Exclusion criteria for RFA treatment were proximity of CLM to major biliary structures and/or bleeding disorders.

### Pre-RFA assessment

Pre-RFA treatment assessments included performance status evaluation, liver function tests, carcinoembryonic antigen (CEA) evaluation, and chest and abdomino-pelvic computed tomography (CT) with contrast agent enhancement at multiple phases (ie, early arterial phase, portal venous phase, and delayed venous phase). The time interval between the last systemic therapy and RFA was 2–4 weeks. Response to systemic therapy was determined according to the Response Evaluation Criteria in Solid Tumors (RECIST) and classified as partial response (PR), stable disease (SD), and progressive disease (PD) [20].

### RFA procedure

RFA was performed percutaneously under conscious sedation (midazolam, 5–20 mg) and analgesia (fentanyl, 50–250 μg) and guided by ultrasonography (US) or computed tomography (CT). All ablations were performed using a 15-gauge needle with 10 expandable hook-shaped electrode tines (LeVeen, Boston Scientific, Natick, MA, USA) connected with a commercially available RF generator (RF 3000, Boston Scientific, Natick, MA, USA) capable of producing 200 W of power. Based on the size of the targeted CLM, the expandable needle electrode (3.0-4.0 cm of exposed tip) was manually inserted into the target CLM. Ablations were performed according to the protocols provided by the manufacturer. Primary end points for a technically successful ablation were ≥2 increases in tissue impedance (roll-off) with an interablation delay of 30s. For CLM with a diameter of ≤1,5 cm, one ablation was performed using a single 3-cm exposed tip electrode. For CLM of >1.5 cm in size, 2–4 overlapping ablations were performed by using 3-4-cm exposed tip electrodes. Irrespective of the use of US or CT-guidance, US and contrast-enhanced US (SonoVue, Bracco, Milan, Italy) was performed routinely after electrode withdrawal, to assess the volume of ablation achieved and to guide additional ablations if the volume of the ablation was considered insufficient in comparison with the pre-RFA tumor size and/or margin. An RFA procedure was considered to be complete when the ablated area encompassed the target CLM by including a ≥0.5 cm ablative margin, as determined by the transient hyperechoic zone (tumor coverage ≥1 cm) and by the lack of contrast enhancement (tumor coverage ≥0.5 cm) at US at the end of the procedure. Needle track ablation was performed to avoid possible seeding of tumor cells and needle track hemorrhage.

### Post-RFA assessment of treatment efficacy

Post-RFA assessments included contrast-enhanced chest and abdomino-pelvic CT imaging and CEA evaluation. Initial post-RFA CT imaging was performed 2–4 weeks after RFA treatment to establish a new baseline, thereafter every 3 months for the first 2 years, and thereafter every 6 months. Each follow-up study was compared to the CT images before RFA and the new baseline CT studies after RFA. Treatment efficacy was determined according to the criteria proposed by the International Working Group on Image-Guided Tumor Ablation [21]. In brief, primary technical success was defined as absence of contrast enhancement in the target CLM on post-RFA CT imaging 2–4 weeks after the RFA procedure. Secondary technical success was defined as absence of contrast enhancement after reablation. Complete ablation was defined as an absence of contrast enhancement in the target CLM ≥3 months after the RFA procedure. Local tumor progression was defined as the development of new focal areas of contrast enhancement at follow-up, either within, or contiguous/adjacent to, the edge of RFA-treated CLM that were previously considered to be completely ablated. All other new contrast-enhancing focal lesions at other intra- and extrahepatic sites were considered new metastases and defined as intra- and/or extrahepatic recurrence.

### Post-RFA assessment of complications

Procedural complications were determined according to the Society of Interventional Radiology (SIR) classification system [22]. Major complications were defined as events associated with substantial morbidity and disability, increasing the level of care and requiring surgical or radiological interventions, blood transfusion, significant medical therapies or longer hospital stay. All other complications were considered minor.

### Data analysis

A chart review of patient demographics, clinical features, tumour-related variables, treatment-related variables including response to each applied systemic regimen, the number of lines and treatment duration of systemic therapy, survival, and the timing and pattern of recurrence after RFA treatment were retrospectively analyzed for each patient. All CT examinations before RFA treatment and for follow-up studies were jointly reviewed by an on-staff abdominal and interventional radiologist.

### Statistical analysis

Categorical and continuous variables are expressed as mean ± standard deviation, median (range), and frequency. Actuarial OS and RFS probabilities were calculated by the Kaplan-Meyer method from the date of first RFA treatment and compared using the log-rank test. Factors subjected to univariate analysis were age, gender, type of primary tumor (colon versus rectum), nodal status of primary tumor (positive versus negative), synchronous CLM (versus metachronous), maximum CLM size, number of CLM, CEA level, and response to systemic therapy. Factors found to be significant on univariate analysis were subjected to multivariate analysis using a Cox proportional hazards model. Estimation of RFS and OS was additionally adjusted for factors found to be present in the patients achieving ≥5-year RFS after RFA treatment. For all analyses, *p* values <0.05 were considered significant. Statistical analyses were carried out using SAS Statistical Software Version 9.1 (SAS, Institute Inc.; Cary, North Carolina, USA).

## Results

### Patient characteristics and chemotherapy details

Clinical features of the study cohort are summarized in Table 1. The study population comprised 57 men and 31 women. Ages ranged from 36 to 85 years (mean, 67.8 years; median, 69 years). At the time of RFA treatment, the median number of CLM was 2.5 (mean: 2.7; range 1–5), which ranged from 0.8 to 5.0 cm in size (mean, 3.1 cm; median, 2.9 cm). With respect to the total exposure to antineoplastic agents, patients received on average 3 lines of systemic therapies before and after RFA treatment (mean, 3.1, range 1–5): a total of 57 patients had received 5-FU monotherapy (65%), 81 (92%) FOLFOX, and 82 (93%) FOLFIRI; bevacizumab and cetuximab had been given to 40 patients (45%) and 16 patients (18%), respectively (Table 1).

**Table 1:** Baseline data for the study cohort

**Characteristic**	**Value**
Patients (n = 88)	
Male, n (%)	57 (65)
Female, n (%)	31 (35)
Mean age ± SD, years	67.8 ± 0.84
Primary tumor	
Colon, n (%)	69 (78)
Rectum, n (%)	19 (22)
Node status	
Positive, n (%)	65 (74)
Negative, n (%)	23 (26)
Colorectal liver metastases (CLM)	
Synchronous, n (%)	22 (25)
Metachronous, n (%)	66 (75)
Mean number ± SD	2.7 ± 0.13
Number of 1–3 CLM, n (%)	67 (76)
Number of 4–5 CLM, n (%)	21 (24)
Mean maximum size ± SD, cm	3.1 ± 0.1
Maximum size ≤ 3 cm, n (%)	51 (65)
Maximum size 3–5 cm, n (%)	27 (35)
CEA levels before RFA, ng/mL	
Mean CEA level ± SD	132.2 ± 16.3
CEA ≤ 100, n (%)	51 (58)
CEA > 100, n (%)	37 (42)
Main cause of unresectability	
Expected liver remnant ≤ 30%, n (%)	19 (22)
Proximity to critical structures, n (%)	22 (25)
Medical comorbidity, n (%)	37 (42)
Patient refusal, n (%)	10 (11)
Systemic therapies before RFA	
Mean number of lines ± SD	1.5 ± 0.07
5-Fluorouracile, n (%)	36 (41)
5-Fluorouracil, Leucovorin, Oxaliplatin (FOLFOX), n (%)	59 (67)
5-Fluorouracil, Leucovorin, Irinotecan (FOLFIRI), n (%)	41 (47)
+ Bevacizumab, n (%)	13 (15)
+ Cetuximab, n (%)	5 (6)
Systemic therapies before and after RFA	
Mean number of lines ± SD	3.1 ± 0.1
5-Fluorouracile, n (%)	57 (65)
5-Fluorouracil, Leucovorin, Oxaliplatin (FOLFOX), n (%)	81 (92)
5-Fluorouracil, Leucovorin, Irinotecan (FOLFIRI), n (%)	82 (93)
+ Bevacizumab, n (%)	40 (45)
+ Cetuximab, n (%)	16 (18)
Response to the immediate systemic therapy before RFA	
Partial remission, n (%)	49 (56)
Stable disease, n (%)	13 (15)
Progressive disease, n (%)	26 (29)
Recurrence after RFA	
Median time to recurrence, months (range)	8 (1–24)
Local tumor progression (RFA-site), n (%)	8 (9)
Intrahepatic recurrence, n (%)	33 (37)
Extrahepatic recurrence, n (%)	14 (16)
Intra-/and extrahepatic recurrence, n (%)	27 (31)
No recurrence, n (%)	6 (7)

*Abbreviations: CEA*, carcinoembryonic antigen; *CLM*, colorectal liver metastases; *RFA*, radiofrequency ablation; *SD*, standard deviation.

Considering chemotherapy details before RFA treatment, a total of 49 patients (56%) underwent RFA after one line, 31 (36%) after two lines, and 9 (8%) after 3 lines of prior systemic therapy. The regimens of each line are detailed in Table 2. Most frequently applied last line regimens before RFA treatment were FOLFOX (40% [35/88] and FOLFIRI (23% [20/88]). There was no difference in tumor response prior to RFA for FOLFOX and FOLFIRI (57% [20/35] vs. 50% [10/20], p >0.05). A total of 49 patients underwent RFA during PR (56%), 13 patients (15%) had SD, and 26 patients (29%) had PD after the immediate preceding systemic therapy prior to RFA treatment (Tables 1 and 2).

**Table 2:** Details of systemic therapy before RFA treatment

**Regimen**	**Line of therapy and regimen administered**				**Response to last line therapy regimen**^**1**^		
	**1st line**	**2nd line**	**3rd line**	**Last line**^**2**^	**PR**	**SD**	**PD**
5-FU, n	30	5	0	17	10	3	4
5-FU + Bevacizumab, n	1	0	0	1	1	0	0
FOLFOX, n (%)	31	19	2	35 (40)	20	5	10
FOLFOX + Bevacicumab, n	1	0	1	2	1	1	0
FOLFOX + Cetuximab, n	3	2	0	5	3	1	1
FOLFIRI, n (%)	18	12	1	20 (23)	10	1	9
FOLFIRI + Bevacizumab, n	3	4	3	8	3	3	2
FOLFIRI + Cetuximab, n	0	0	0	0	0	0	0
Total number (%)	88 (100)	40 (45)	8 (9)	88 (100)	49	13	26

*Abbreviations: FOLFIRI*, 5-fluorouracil,leucovorin, irinotecan; *FOLFOX*, 5-fluorouracil, leucovorin, oxaliplatin; *RFA*, radiofrequency ablation.

^1^RECIST-defined response to the last line regimen before RFA treatment. ^2^49 patients (56%) received RFA after 1st line, 31 (36%) after 2nd line, and 8 (9%) after 3rd line therapy.

### Outcome and complications after RFA treatment

Primary technical success was achieved in 93.6% (221 of 236) CLM. Of the CLM, 6.4%% (15 of 236) required early reablation due to residual enhancing tumor on follow-up CT scans (≤2-4 weeks). Secondary technical success was obtained in 100% (15 of 15). At a median follow-up of 99 months (range 63–134 months) following RFA treatment, a total of 82 patients (93%) had developed disease recurrence. The frequencies of first-site recurrences are shown in Table 1. Six patients (7%, [6/88]) remained recurrence-free >5-years following RFA treatment (Table 3). There were no procedure-related deaths. Adverse events related to the procedure were observed in 10.2% of patients (9 of 88). Two patients (2.3%) developed major complications (2× infected biloma requiring drainage and antibiotic therapy). The remaining 7 patients had one or more self-limiting minor complications (5× fever, 2× pain, 2× pleural effusions, and 1× small intrahepatic hematoma).

**Table 3:** Characteristics of patients achieving ≥5-year survival without disease recurrence after systemic therapy plus RFA for liver-only CLM

**Patient**	**Systemic therapy**			**Clinical features**		
	**Response**^**1**^	**No. of lines**	**Regimen(s) administered**	**Number of CLM**	**Size**^**2 **^**of CLM**	**CEA level (ng/ml)**
1	Partial remission	1	FOLFIRI	1	2.5 cm	12
2	Partial remission	2	FOLFOX, FOLFIRI	1	2.1 cm	23
3	Partial remission	1	FOLFOX	2	2.2 cm	13
4	Partial remission	2	FOLFIRI + Bevacizumab, FOLFOX + Cetuximab	3	3.0 cm	114
5	Partial remission	1	FOLFIRI + Bevacizumab	2	3.1 cm	57
6	Partial remission	1	FOLFOX	1	2.7 cm	4

*Abbreviations: CEA*, carcinoembryonic antigen; *CLM*, colorectal liver metastases; *FOLFIRI*, 5-fluorouracil, leucovorin, irinotecan; *FOLFOX*, 5-fluorouracil, leucovorin, oxaliplatin; *RFA*, radiofrequency ablation.

^1^RECIST-defined response to the immediate preceding regimen before RFA treatment. ^2^after decrease of ≥30% in size following the immediate preceding regimen before RFA treatment.

### Univariate and multivariate analysis

**Figure 1:**
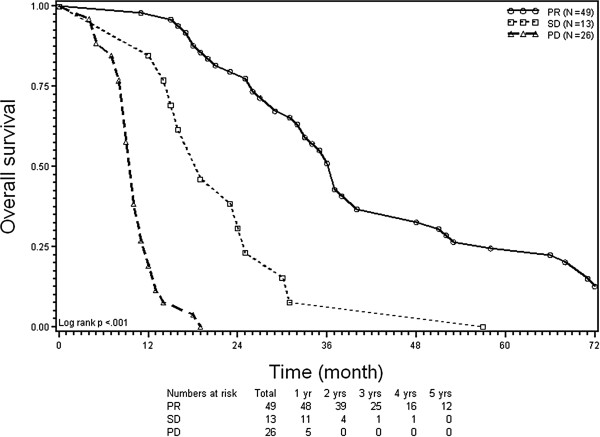
**Kaplan-Meier plots of overall survival (OS).** Stratification according to response to chemotherapy before radiofrequency ablation of colorectal liver metastases. PR = partial remission; SD = stable disease; and PD = progressive disease.

**Figure 2:**
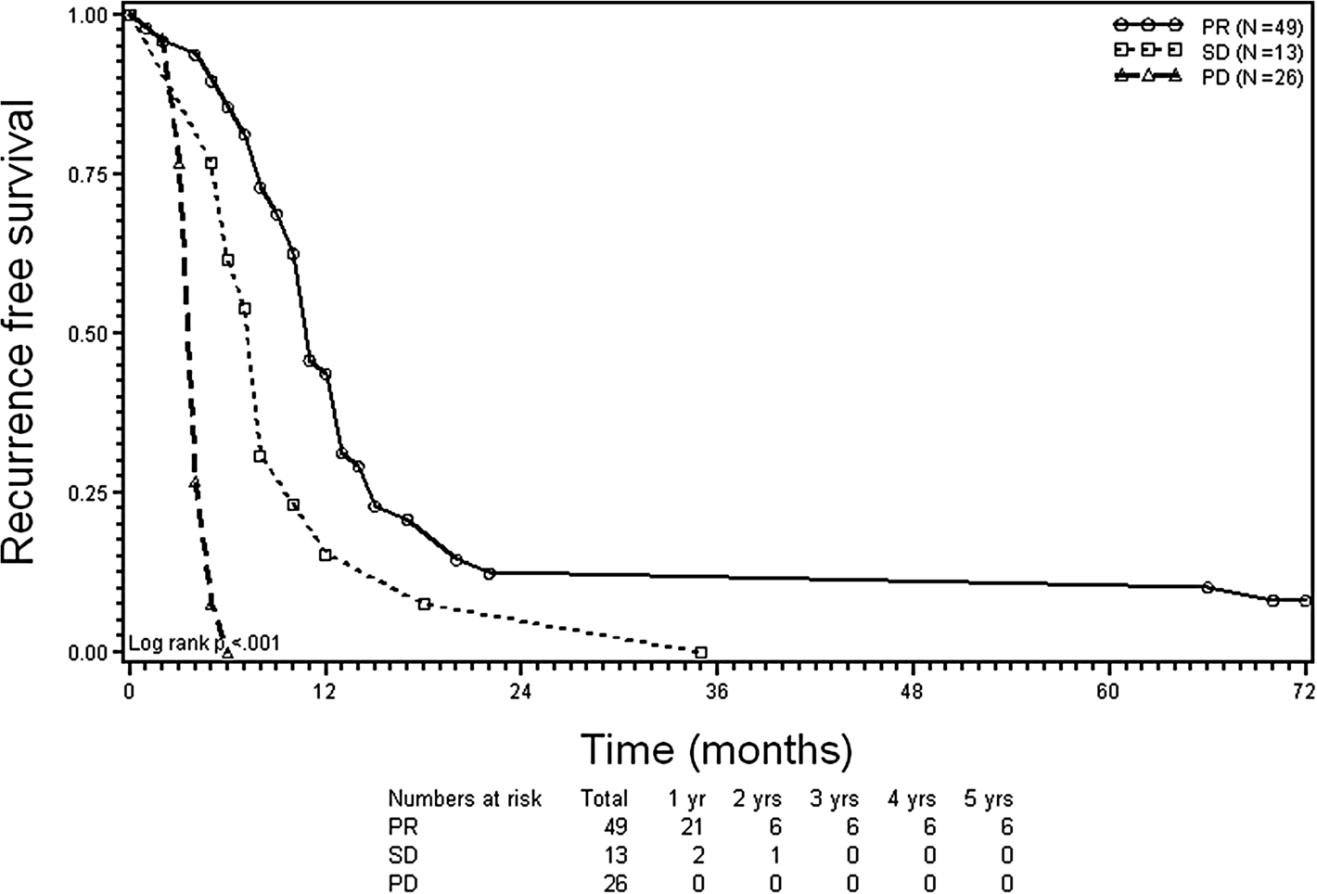
**Kaplan-Meier plots of recurrence-free survival (RFS).** Stratification according to response to chemotherapy before radiofrequency ablation of colorectal liver metastases. PR = partial remission; SD = stable disease; and PD = progressive disease.

At univariate analysis, four factors significantly (p < 0.05, respectively) negatively influenced both OS and RFS (Table 4): no response to the immediate pre-RFA systemic therapy (Figures 1 and 2), lesion size >3 cm, number of ≥4 CLM, and CEA level ≥100 ng/ml. At multivariate analysis, independent negative prognostic factors for OS and RFS were PD before RFA (hazard ratio (HR) 5.46; *p* < 0.0001; and HR 6.46; *p* < 0.0001), ≥4 CLM HR 3.13; *p* = 0.0005; and HR 1.77; *p* = 0.039), and CEA level ≥100 ng/ml (HR 1.67; *p* = 0.032; and HR 1.67; *p* = 0.044). It should be noted that there were no patients achieving 5-year RFS with CLM of >3 cm in size (Table 4).

**Table 4:** Univariate and multivariate analysis of variable factors for overall survival and recurrence-free survival

		**Overall survival**						**Recurrence-free survival**					
		**Univariate analysis**			**Multivariate analysis**			**Univariate analysis**			**Multivariate analysis**		
**Variable**	**No. of patients**	**Median months (95% CI)**	**5-years, % (95% CI)**	**p-value**	**p-value**	**HR**	**HR 95% CI**	**Median months (95% CI)**	**5-years % (95% CI)**	**p-value**	**p-palue**	**HR**	**HR 95% CI**
Age				0.8381						0.4263			
≤ 60 years	16	19 (14–37)	8.3 (0.6-30.2)					8 (5–13)	13.3 (2.2-34.6)				
> 60 years	72	25 (18–31)	12.4 (5.8-21.5)					8 (6–10)	5.6 (1.8-12.5)				
Gender				0.6264						0.8117			
Male	57	23 (17–30)	10.5 (3.9-28.4)					7 (5–11)	7.1 (2.3-15.8)				
Female	31	24 (15–37)	14.3 (4.6-29.3)					9 (6–11)	6.5 (1.1-18.6)				
Primary tumor				0.1872						0.7773			
Colon	69	19 (16–27)	8.6 (3.3-17.2)					8 (6–10)	5.9 (1.9-13.2)				
Rectum	19	33 (23–58)	23.7 (7.2-45.5)					7 (4–13)	10.5 (1.8-28.4)				
Node status				0.8512						0.4566			
Positive	65	25 (19–32)	11.4 (5.0-2.8)					8 (6–10)	6.2 (2.0-13.8)				
Negative	23	18 (14–33)	13.0 (2.6-32.3)					10 (5–13)	9.1 (1.6-25.1)				
CLM				0.9861						0.2889			
Synchronous	22	20 (18–35)	9.1 (1.6-25.1)					10 (7–13)	4.5 (0.3-18.9)				
Metachronous	66	26 (16–32)	12.4 (5.4-22.4)					7 (5–10)	7.7 (2.8-15.8)				
CEA level before RFA				0.0189	0.0325					0.0062	0.0442		
≤ 100 ng/ml	51	29 (19–37)	15.3 (6.7-27.0)					10 (8–12)	9.8 (3.6-19.7)				
> 100 ng/ml	37	15 (11–25)	6.9 (1.4-19.1)			1.672	1.044-2.678	6 (4–7)	2.8 (0.2-12.4)			1.637	1.013-2.647
Maximum size of CLM				0.0001	0.0939					<0.0001	0.0771		
≤ 3 cm	61	30 (23–36)	15.3 (7.2-26.1)					10 (8–11)	9.8 (4.0-18.8)				
3-5 cm	27	12 (10–19)	3.8 (0.3-16.4)			1.548	0.928-2.581	5 (4–6)	0			1.600	0.950-2.694
Number of CLM				<0.0001	<0.0001					0.0093	0.0389		
1-3	67	27 (21–36)	15.6 (7.8; 25.9)					8 (7–11)	9.0 (3.6-17.2)				
4-5	21	12 (10–18)	0			3.128	1.771-5.526	5 (4–10)	0			1.771	1.029-3.048
Response to systemic therapy before RFA				<0.0001	0.0005					<0.0001	<0.0001		
Partial remission	49	37 (32–48)	21.6 (10.9-34.7)					11 (10–13)	12.5 (5.1-23.4)				
Stable disease	13	19 (15–25)	0					8 (6–10)	0				
Progressive disease	26	10 (9–11)	0			5.456	2.289-13.005	4 ()	0			6.458	2.644-15.78

*Abbreviations: 95% CI*, 95% confidence interval; *CEA,* carcinoembryonic antigen; *CLM*, colorectal liver metastases; *HR*, Hazard ratio; *RFA,* radiofrequency ablation.

### Scoring system for predicting outcome

**Figure 3:**
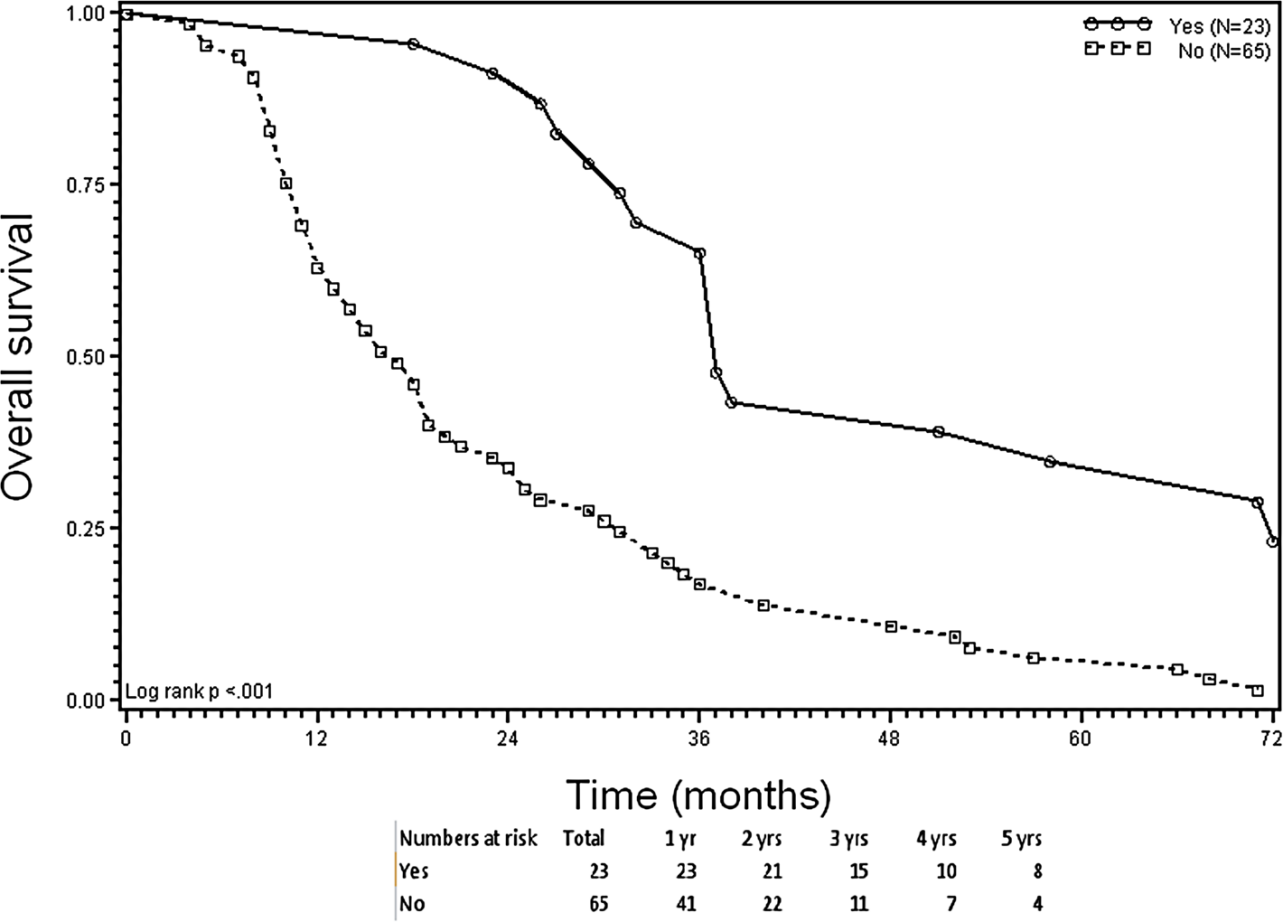
**Kaplan-Meier plots of overall survival (OS).** Stratification according to the presence (score 4) versus partial or complete absence (score 0–3) of 4 criteria before radiofrequency ablation of colorectal liver metastases. Criteria: (1) response to prior systemic therapy; (2) ≤3 CLM; (3) ≤3 cm lesion size; and (4) carcinoembryonic antigen level ≤100 ng/mL.

**Figure 4:**
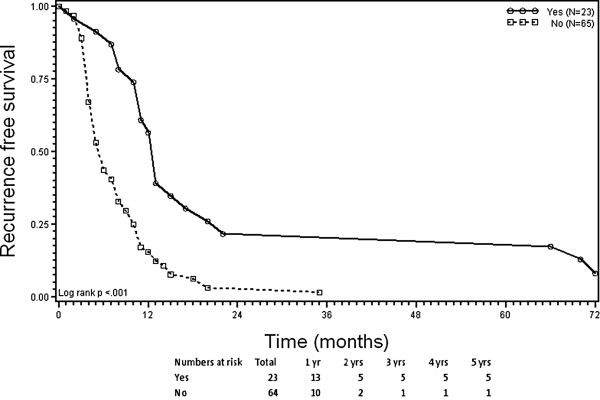
**Kaplan-Meier plots of recurrence-free survival (RFS).** Stratification according to the presence (score 4) versus partial or complete absence (score 0–3) of 4 criteria before radiofrequency ablation of colorectal liver metastases. Criteria: (1) response to prior systemic therapy; (2) ≤3 CLM; (3) ≤3 cm lesion size; and (4) carcinoembryonic antigen level ≤100 ng/mL.

To address the issue of patient selection, we developed a prognostic scoring system for quantifying the probabilities of OS and RFS of individual patients in the pre-RFA setting. The scoring system was developed in three steps. First, to determine criteria most strongly associated with long-term disease control, we analyzed the characteristics of the 6 patients obtaining 5-year RFS. Uniformly, those patients presented with ≤3 CLM of ≤3 cm maximum size after effective systemic therapy (regardless of the regimen applied), and 5 of the 6 patients had CEA level ≤100 ng/ml (Table 3). Second, the derivation of our prognostic scoring system started from these four criteria and their reference categories (objective response, ≤3 CLM, ≤3 cm, and CEA ≤100 ng/ml) because of their strong association with long-term disease control in our data set (Table 5). By assigning one point to each criterion, we defined a scoring scale (score 0,1,2,3, or 4; indicates the sum of the presence of zero, one, two, three, or four criteria) and formed subgroups for scores 1,2, and 3 (representing the combination of the presence or absence of criteria). Third, calculation of Kaplan-Meier curves for OS and RFS adjusted on these scores and subgroups created a scoring system providing estimates for median and 5-year probabilities of OS and RFS based upon the number and combination of the presence or absence of the four criteria (Table 6). The score also separated two fundamental subsets (score 4 vs. scores 0–3) with significantly different OS and RFS curves across the entire cohort (p < 0.001). Patients scoring 0–3 distributed along a wide range of median times for OS (16–44 months) and RFS (4–11 months); the 5-year probabilities for OS (0-27%) and RFS (0-9%) were relatively low (Table 6). Patients scoring 4 (n = 23) had significantly higher median times for OS (46 months [95% CI 40–76 months]) and RFS (13 months [95% CI 11–17 months]) and significantly higher 5-year probabilities for OS (39% [95% CI 20-58%] and RFS (22% [95% CI 8-40%]) compared to patients scoring 0–3 (p < 0.001, Figures 3 and 4, Table 6).

**Table 5:** Criteria used to build a prognostic score for patient selection for RFA treatment of liver-only CLM in the context of effective modern-agent systemic therapies

**Variable**	**Reference category**	**Points**
Systemic therapy	Objective response^1^	1
Number of CLM	≤ 3 CLM	1
Maximum size of CLM	≤ 3 cm^2^	1
CEA level	≤ 100 ng/mL	1

*Abbreviations*: *CEA*, carcinoembryonic antigen; *CLM*, colorectal liver metastases; *RFA*, radiofrequency ablation.

^1^RECIST-defined response to the immediate preceding systemic therapy regimen before RFA treatment. ^2^after decrease of ≥30% in size following the immediate preceding systemic therapy regimen before RFA treatment.

**Table 6:** **Prognostic scoring system providing probabilities of survival outcomes based on presence (+) or absence (-) of four criteria for improving patient selection for RFA treatment of liver-only CLM in the era of effective systemic therapies**^**1**^

		**Clinical criteria**				**Overall survival**		**Recurrence-free survival**	
**Score**^**2**^	**Subgroup**^**3**^	**Response**^**4 **^**PR**	**No. of CLM ≤ 3**	**Size of CLM**^**5**^ **≤ 3 cm**	**CEA value ≤ 100 ng/mL**	**median (months)**	**5-Years (%)**	**median (months)**	**5-years (%)**
**0**		**-**	**-**	**-**	**-**	16	0	4	0
**1**	**1A**	**-**	**-**	**-**	**+**	17	0	3	0
	**1B**	**-**	**-**	**+**	**-**	19	0	6	0
	**1C**	**-**	**+**	**-**	**-**	19	0	4	0
	**1D**	**+**	**-**	**-**	**-**	31	0	11	0
**2**	**2A**	**-**	**-**	**+**	**+**	18	0	4	0
	**2B**	**-**	**+**	**-**	**+**	24	0	5	0
	**2C**	**+**	**-**	**-**	**+**	no	no	no	no
	**2D**	**-**	**+**	**+**	**-**	19	0	4	0
	**2E**	**+**	**-**	**+**	**-**	33	0	9	0
	**2 F**	**+**	**+**	**-**	**-**	36	11	10	0
**3**	**3A**	**-**	**+**	**+**	**+**	27	7	5	0
	**3B**	**+**	**+**	**-**	**+**	44	15	9	0
	**3C**	**+**	**-**	**+**	**+**	40	0	11	0
	**3D**	**+**	**+**	**+**	**-**	44	27	11	9
**4**		**+**	**+**	**+**	**+**	46	39	13	22

*Abbreviations*: *CEA,* carcinoembryonic antigen; *CLM*, colorectal liver metastases; *no*, not observed; *PR,* partial remission; *RFA*, radiofrequency ablation.

^1^outcome probabilities are based on adjusted Kaplan-Meyer calculations in this study cohort (n = 88). ^2^score indicates the sum of present criteria. ^3^subgroups represent combinations of present and absent criteria in patients scoring 1–3. ^4^RECIST-defined response to the immediate preceding systemic therapy regimen before RFA treatment. ^5^after decrease of ≥30% in size following the immediate preceding systemic therapy regimen before RFA treatment.

## Discussion

The clinical benefit from RFA as a treatment for CLM is still being debated, and currently no criteria exist to select patients for its use in the context of effective modern-agent therapies. The results of this study indicate that multimodality treatment of liver-limited CLM, consisting of effective systemic therapy plus RFA, may offer the chance to obtain long-term disease control for well-selected patients. According to our findings, a scoring system based on four criteria (response to systemic therapy, ≤3 CLM, ≤3 cm lesion size, and low CEA level) may facilitate selection of patients for RFA treatment for whom best 5-year survival outcomes could be obtained. Patients fulfilling all four criteria (score 4) had significantly higher probabilities for OS and RFS at 5 years after RFA treatment (39% and 22%, respectively) compared to patients scoring 0–3 (0-27% and 0-9%, respectively).

CEA level, number and size of CLM are well-known prognostic factors in patients with RFA-treated CLM [12-18], reflecting the tumor burden and limitations in controlling CLM of ≥3 cm in size using RFA [10,11,23]. Specific to the present study, we found that response to systemic therapy was the most powerful prognostic factor in our RFA cohort, emphasizing the integration of response into the decision making for RFA treatment. A key feature of the prognostic score formulated in this study is the combination of outcome indicators related to both "tumor response" and "tumor burden". This goes beyond establishing associations between single factors and outcome and, instead, gives estimates of outcome for medians and 5-year probabilities of OS and RFS that clinicians can use to guide their decision for RFA treatment of CLM.

Our proposed scoring system is simple, based on four widely available criteria, and usable in the context of effective modern-agent therapies. Although the relative impact for recurrence and outcome varied somewhat for these four criteria, we decided to assign each criterion one point for simplicity and thus enhanced clinical applicability. This design is supported by our data, which show that the scoring system allows for a good prognostic discrimination and selection of patients for RFA treatment. Fundamentally, the total score assigning each criterion one point defines two subgroups: patients who may (score 4) and patients who may not (scores 0–3) potentially obtain 5-year RFS after RFA for CLM. Related to subgroups for scores 1, 2, or 3, which represent the combination of presence or absence of the criteria, patients distribute along a wide range of different median OS and RFS times (Table 6). This reflects the different relative prognostic impact of the underlying criteria. Thus, the scoring system may identify both patients potentially achieving long-term disease control and patients achieving a clinically relevant recurrence-free time without toxicity following RFA of CLM.

The low use of targeted agents in our study reflects their limited availability during the study period (2002–2007) [3]. This excluded a meaningful analysis of new time-related and/or non-size based modified RECIST criteria in our response evaluation of systemic therapies prior to RFA treatment [24]. However, modified RECIST criteria can add prognostic information to conventional size-based RECIST response categories used in this study when assessing response to targeted agents such as cetuximab [25] or bevacizumab [26]. Therefore, further refinement of the RECIST methodology by modified RECIST criteria would rather enhance than reduce the clinical usefulness of our prognostic score, particularly with the new targeted agents now available for routine use [7].

Almost one-fourth (22%) of our RFA-treated responders to systemic therapy with a low residual tumor burden (≤3 CLM, ≤3 cm, low CEA value) achieved 5-year RFS. The median RFS of 13 months is also clinically relevant and underscores a beneficial effect from RFA within this subgroup. Interestingly, our long-term results are consistent with reported rates for 5-year RFS (9-32%) after RFA treatment in particular subgroups with solitary and/or small (≤3 cm) CLM [15,17] and/or CLM responding to chemotherapy [16].

For several reasons, a long-term result with a 5-year RFS rate of 22% would probably be unrealizable in a similar group of patients when treated with systemic therapy alone. First, no study has yet reported the possibility of 5-year RFS after systemic therapy of CLM alone. Second, the best currently available evidence, the randomized phase II CLOCC trial, supports a benefit with respect to disease control by adding RFA to chemotherapy even for patients with up to 9 CLM [19]. Thirdly, local response to systemic therapy is rarely complete [4-6], and RFA can improve local disease control. Because RFS captures the time-related effect of both local and distant disease control, it seems likely that RFA contributed to the 5-year RFS observed.

For patients with liver-only CLM responding to systemic therapy, reported 5-year rates of OS (≤15%, [27,28]) are lower compared to that (39% [95% CI 20-58%]) seen in a subset of our additionally RFA-treated responders. It could be argued that patient selection bias is responsible for this difference in OS. However, one cannot absolutely exclude an impact on OS from additional local therapy by RFA, as shown for patients with CLM resected after response to chemotherapy [2,27,28]. Although resection series and RFA series like ours are hardly comparable, a similar finding is that response to preoperative chemotherapy, number of ≤3 CLM, size of ≤3 cm, and low CEA level also represent powerful predictors of postoperative outcome [1,28,29]. The long-term result, however, seen in our RFA series is not as good as reported after resection of CLM responding to chemotherapy, which results in rates for 5-year OS of 33-64% [1,2,28].

A limitation of this study is the retrospective nature of observing treated patients including a methodically non-avoidable selection bias. Our observations were also limited to a single-institutional experience. Additionally, comparison with a group receiving chemotherapy alone was not performed, because it was not possible to select a population of patients receiving chemotherapy only with comparable disease extent.

## Conclusions

This study provides long-term outcome data indicating the potential of 5-year survival without disease recurrence for a defined subgroup of patients receiving RFA for liver-only CLM. The four criteria defining this subgroup (response to systemic therapy, number of CLM, size of CLM, and CEA level) may provide a clinical score for predicting outcome and improving patient selection for RFA treatment of CLM in the era of effective systemic therapies. This score may also aid in the interpretation and comparison of outcome data and conduct of clinical trials, including defining a cohort that could be tested in a randomized fashion. Further prospective multicenter studies are needed to confirm our findings, ideally designed as randomized trials.

## Abbreviations

CEA: Carcinoembryonic antigen; CLM: Colorectal liver metastases; CT: Computed tomography; OS: Overall survival; PD: Progressive disease; PR: Partial response; RFA: Radiofrequency ablation; RFS: Recurrence-free survival; SD: Stable disease; US: Ultrasonography.

## Competing interests

The authors declare that they have no conflict of interest.

## Authors’ contributions

AS drafted the manuscript, carried out the conception and design, analysis and interpretation of data. KJO participated in the conception and design of the study and helped to draft the manuscript. HW participated in the design of the study and performed the statistical analysis and interpretation of data. HK participated in the data acquisition and the statistical analysis of data and helped to draft the manuscript. MD participated in the analysis and interpretation of data, participated in the design of the study, and revised the manuscript critically. All authors read and approved the final manuscript.

## Pre-publication history

The pre-publication history for this paper can be accessed here:

http://www.biomedcentral.com/1471-2407/14/500/prepub
